# Body composition shapes cardiometabolic risk in children with multifactorial dyslipidemia

**DOI:** 10.3389/fnut.2025.1717055

**Published:** 2026-01-15

**Authors:** Gaya Hamou, Asaf Ben Simon, Michal Yackobovitch-Gavan, Hagar Interator, Adar Lopez, Ronit Lubetzky, Avivit Brener, Yael Lebenthal

**Affiliations:** 1 The Institute of Pediatric Endocrinology, Diabetes and Metabolism, Dana-Dwek Children's Hospital, Tel Aviv Sourasky Medical Center, Tel Aviv, Israel; 2 Gray School of Medicine, Faculty of Medical and Health Sciences, Tel Aviv University, Tel Aviv, Israel; 3 Department of Epidemiology and Preventive Medicine, School of Public Health, Gray Faculty of Medicine and Health Sciences, Tel Aviv University, Tel Aviv, Israel; 4 The Nutrition and Dietetics Unit, Tel Aviv Sourasky Medical Center, Tel Aviv, Israel; 5 Department of Pediatrics, Dana-Dwek Children's Hospital, Tel Aviv Sourasky Medical Center, Tel Aviv, Israel

**Keywords:** bioelectrical impedance, cardiovascular disease risk, dyslipidemic atherogenic index, fat mass, multifactorial dyslipidemia, muscle-to-fat ratio, physical activity

## Abstract

**Introduction:**

Childhood-onset multifactorial dyslipidemia is associated with obesity and early cardiometabolic risk, yet weight status alone may not reflect abnormalities in body composition. We aimed to examine associations between body composition, physical activity, and cardiometabolic markers in this population.

**Methods:**

This observational study included 101 children (mean age 13.8 ± 3.2 years) referred to a tertiary lipid clinic. Body composition was assessed by bioelectrical impedance analysis. Individuals were stratified by fat mass z-score (<2 vs. ≥2) to compare clinical characteristics. Cardiometabolic markers included blood pressure percentiles, the homeostasis model assessment of insulin resistance (HOMA-IR), and the triglyceride-to-high-density lipoprotein cholesterol (HDL-C) ratio.

**Results:**

Most participants (67%) had obesity, high fat mass (median z-score 3.51), low muscle-to-fat ratio (z-score–1.62), and low physical activity (2 h/week). Those with fat mass z-scores ≥2 had higher blood pressure percentiles, triglycerides, HOMA-IR, and triglyceride-to-HDL-C ratios, and lower muscle-to-fat ratios (all *p* < 0.05). Fat mass z-score correlated with all cardiometabolic markers. In multivariable models, lower muscle-to-fat ratio (β = −15.0), lower physical activity (β = −3.1), and female sex (β = 9.9) were independently associated with higher diastolic blood pressure percentiles.

**Discussion:**

In pediatric multifactorial dyslipidemia, both adiposity and muscle-to-fat imbalance contribute to early cardiometabolic risk and may improve risk stratification.

## Introduction

Multifactorial dyslipidemia is a common lipid disorder that results from the interplay of genetic predisposition, environmental influences, and lifestyle factors. It is typically characterized by elevated triglyceride levels and/or reduced high-density lipoprotein cholesterol (HDL-C) concentrations (1). This condition frequently coexists with obesity, suboptimal dietary patterns, physical inactivity, and other modifiable cardiovascular risk factors (2). Multifactorial dyslipidemia in the pediatric population is strongly associated with obesity and insulin resistance (3). The American College of Cardiology and the American Heart Association note its high prevalence among children and adolescents with obesity and its contribution to increased long-term cardiovascular and metabolic risk (4).

Elevated triglyceride and low HDL-C concentrations contribute to the development of atherosclerosis in children with multifactorial dyslipidemia. High triglyceride concentrations lead to the formation of small, dense low-density lipoprotein (LDL) particles, which are more atherogenic and prone to oxidation (5). Concurrently, low HDL-C levels impair reverse cholesterol transport, a key mechanism for removing excess cholesterol from arterial walls. This lipid imbalance, which is frequently observed in children living with obesity, is associated with increased arterial stiffness and insulin resistance, thereby heightening the risk for early cardiovascular disease (6, 7). The dyslipidemic atherogenic index, defined as the triglyceride (TG): HDL-C ratio, has emerged as a useful marker for identifying children at risk for hypertension (8). A growing body of evidence has suggested that this ratio is a more reliable predictor of overall cardiometabolic risk than individual lipid parameters (9). Mechanistic studies further support this association, showing that hepatic transcriptional regulators such as Krüppel-like factor 10 (KLF10) protect against lipid dysregulation and steatohepatitis by maintaining HNF4α-dependent cholesterol and bile-acid metabolism (10).

Given its role in improving lipid profiles and reducing cardiometabolic risk, physical activity is considered a central component of the prevention and management of dyslipidemia in children. The World Health Organization recommends that children and adolescents (5–17 years of age) engage in an average of 60 min of moderate-to-vigorous intensity aerobic physical activity daily to promote overall health, including improved cardiometabolic function, body composition, bone strength, and cognitive performance (11). The health benefits of exercise are primarily determined through total daily energy expenditure, often quantified by metabolic equivalent tasks (METs) (12).

In addition to physical activity, body composition plays a role in shaping cardiometabolic risk in youth, particularly in the context of dyslipidemia and obesity. Our group previously demonstrated the predictive value of muscle-to-fat ratio (MFR) z-scores in identifying children and adolescents at increased risk for cardiovascular disease (CVD) (13–15). An imbalance between muscle and fat tissue—specifically, low muscle mass relative to fat mass—was associated with a higher likelihood of early-onset cardiometabolic complications in youth with overweight or obesity (13, 15). Recent findings from a population-based study in Austria supported this relationship, showing that children and adolescents with high fat mass and low muscle mass face greater risk for developing dyslipidemia than those with abnormalities in either compartment alone (16). These findings suggest that assessment of body composition may enhance screening efforts for lipid disorders in youth.

Importantly, childhood dyslipidemia, even when it resolves by adulthood, has been identified as a predictor of adult carotid plaque, underscoring the long-term implications of early lipid abnormalities (17). In 2018, the Pediatric Endocrine Institute implemented a comprehensive metabolic risk assessment for all referred patients, including the evaluation of body composition by means of bioimpedance analysis (BIA) as part of routine standard care (14).

The aim of the present study is to explore the associations between weight status, body composition, physical activity, lipid profiles, and cardiometabolic outcomes in pediatric patients with multifactorial dyslipidemia, a relationship that remains inadequately characterized in the current literature. We hypothesize that a lower muscle-to-fat ratio, measured by BIA, is independently associated with a more adverse cardiometabolic risk profile in pediatric multifactorial dyslipidemia.

## Methods

### Study design and population

This retrospective observational study included pediatric patients aged 5–18 years who were referred to the Lipid Clinic at a tertiary medical center between January 2023 and December 2024. Eligible participants were identified by querying the institutional BIA database for individuals diagnosed with dyslipidemia, with analyses limited to data from their initial evaluation. Relevant clinical and laboratory data were extracted from electronic medical records. Inclusion criteria required available body composition measurement and lipid profile results, with no prior exposure to lipid-lowering therapy. A total of 67 individuals were excluded for the following reasons: prolonged steroid therapy (*N* = 20), type 1 diabetes (*N* = 14), lipodystrophy (*N* = 7), gender dysphoria on gender-affirming hormone therapy (*N* = 7), genetic syndromes (*N* = 6), renal disease (*N* = 5), malignancies (*N* = 3), type 2 diabetes (*N* = 2), Graves’ disease (*N* = 1), Cushing’s disease (*N* = 1), and antiretroviral therapy for HIV infection (*N* = 1). The final cohort comprised 101 children and adolescents with multifactorial dyslipidemia (36 boys).

### Clinical evaluation

The study was approved by the Ethics Committee of the Tel-Aviv Sourasky Medical Center (TLV-0734-23) according to the Helsinki Declaration. The requirement for informed consent from parents or legal guardians, as well as assent from participants aged 16 years and older, was waived by the Institutional Review Board due to the retrospective design of the study and the use of de-identified data. The data were handled in accordance with the principles of Good Clinical Practice. As part of standard clinical care, all participants underwent a structured medical interview (anamnesis) and physical examination. Clinical assessment included standardized measurements of height by means of a Harpenden stadiometer (Holtain Ltd., Crosswell, UK), and weight and body composition by means of BIA, with individuals dressed in light clothing. Blood pressure (BP) was measured by a registered pediatric nurse using the Welch Allyn Vital Signs Monitor VSM 300 (Welch Allyn, Inc., Beaverton, OR) and an appropriately sized cuff. BP was measured in the seated position after a period of rest, using an appropriately sized cuff, in accordance with pediatric guidelines (18). Three consecutive readings were obtained in the seated position after a period of rest, and the average of the last two measurements was used for analysis. A full physical examination, including pubertal staging, was performed. Cardiovascular risk stratification was based on a composite of weight status, body composition parameters, BP levels and lipid profile.

### Body composition analysis

Body composition was assessed using BIA with the Tanita MC-780 MA Body Composition Analyzer and GMON Professional Software, as previously described (19). This method has been validated for accuracy, reproducibility, and reliability in pediatric populations (19). Measurements were conducted during routine morning clinic visits (between 8:00 and 11:00 a.m.), preferably in a fasting state and not following recent intense physical activity. The analysis provided whole-body and segmental assessments (trunk, upper, and lower limbs) of fat and muscle. Calculated parameters included the trunk-to-appendicular fat ratio (trunk fat mass divided by appendicular fat mass), appendicular skeletal muscle mass (ASMM, sum of muscle mass in all four limbs), and muscle-to-fat ratio (ASMM divided by fat mass). Z-scores for fat mass and MFR were calculated based upon sex- and age- specific pediatric BIA reference values (20). Fat mass z-scores between −2 and +2 were considered within normal limits, consistent with conventional reference standards used for other anthropometric z-scores in the pediatric population. Individuals were stratified into two groups: those with fat mass z-scores ≥2 and those with z-scores <2.

### Assessment of physical activity levels

To assess physical activity, we employ an open-ended question approach in which patients are requested to describe the types of physical activities in which they engage, along with the frequency (times per week) and duration (hours per week) of carrying out these activities. Physical activity intensity was categorized according to METs, which assess energy expenditure in relation to resting metabolism. Light-intensity activities correspond to 1.6 to 2.9 METs, moderate-intensity activities range from 3.0 to 5.9 METs, and vigorous-intensity activities are those at from 6.0 METs and higher (21).

### Biochemical analysis

The laboratory evaluation included fasting serum concentrations of glucose, insulin, glycated hemoglobin (HbA1c), C-reactive protein (CRP), alanine aminotransferase (ALT), thyroid-stimulating hormone (TSH), and a fasting lipid profile comprising total cholesterol, triglycerides, HDL-C, non-HDL-C, and LDL-C. Insulin resistance was estimated with the homeostatic model assessment of insulin resistance (HOMA-IR), calculated as: fasting insulin (IU/ml) × fasting glucose (mg/dl) ÷ 405 (22). The threshold value of the HOMA-IR result for identifying insulin resistance was ≥ 1.7 for pre-pubertal children (23), > 3.22 for pubertal adolescents, and > 2.91 for post-pubertal adolescents (24). Lipid profile z-scores were derived based upon sex- and age-specific Israeli pediatric reference data (25). The dyslipidemic atherogenic index (TG:HDL-C) was calculated as a surrogate marker of cardiovascular disease risk. Given that LDL-C is calculated using triglyceride-based formulas and may be unreliable in the setting of hypertriglyceridemia, we report non-HDL-C in all analyses, as it is a validated marker of atherogenic burden that includes all atherogenic lipoproteins.

### Data collection

All hospital medical records were maintained electronically, with access to pre-referral laboratory data retrieved from community clinics and national healthcare databases through interoperable health information systems. Sociodemographic information, perinatal and medical history were obtained from those records. Clinical status, anthropometric measurements, vital signs, pubertal staging, and laboratory results were collected at the time of the first BIA assessment. Socioeconomic position (SEP) cluster, which is determined based upon home address using the Israel Central Bureau of Statistics’ classification of statistical areas, is scored from 1 to 10 and categorized as low (1–4), medium (5–7), or high (8–10) (26).

Birth weight z-scores were calculated by PediTools Electronic Growth Chart Calculators (27). Body mass index (BMI) was calculated as weight (kg) divided by height squared (m^2^). BMI z-scores were determined according to the Centers for Disease Control and Prevention 2000 growth charts using sex-and age- specific reference data (28). Weight status was defined by BMI z-scores as follows: underweight (z-score ≤1.65), overweight (1.04 ≤ z-score <1.65), and obesity (z-score ≥1.65) (29). Systolic and diastolic BP percentiles were calculated by a sex-, age- and height-based pediatric BP calculator (30). Pubertal staging was determined according to Tanner criteria based upon testicular volume in boys and breast development in girls (31, 32).

### Statistical methods

All analyses were performed using the Statistical Package for the Social Sciences software version 29 (SPSS Inc., Chicago, IL, USA). All statistical tests were two-sided. The Kolmogorov–Smirnov test or the Shapiro–Wilk test was used to assess the normality of continuous data. Data were expressed as means ± standard deviations for normally distributed variables and as medians with interquartile ranges [IQR] for skewed distributions. Comparisons between individuals with a fat mass z-score <2 and ≥2 were conducted using Pearson’s chi-square tests for categorical variables. Independent samples t-tests or Mann–Whitney U tests were used for numerical variables with normal or skewed distributions, respectively. Spearman’s correlation tests were used to assess the relationships between two continuous variables with skewed distributions. Multivariable linear regression models were employed to examine contributory factors to cardiometabolic outcomes, including systolic and diastolic BP percentiles, HOMA-IR, and the TG:HDL-C ratio. Separate models were constructed for each outcome, with sex, age, and physical activity levels included as covariates, along with one explanatory variable of interest (either the BMI z-score, fat mass z-score, or MFR z-score). To address potential concerns regarding the causal pathway between body composition and blood pressure, additional analyses were performed without adjustment for physical activity to preserve the total effect of body composition on BP outcomes, as physical activity may lie on the causal pathway (adiposity ↔ activity → BP). Nested multivariable linear regression models were employed to examine contributory factors to cardiometabolic outcomes, including systolic and diastolic BP percentiles, HOMA-IR, and the TG:HDL-C ratio. For each outcome, three sequential models were constructed: Model A: Sex and age only; Model B: Sex, age, and fat mass z-score; Model C: Sex, age, fat mass z-score, and MFR z-score. Standardized beta coefficients and change in R^2^ (ΔR^2^) were reported to assess the incremental contribution of each variable. A *p* value of ≤0.05 was considered statistically significant.

## Results

**Figure 1:**
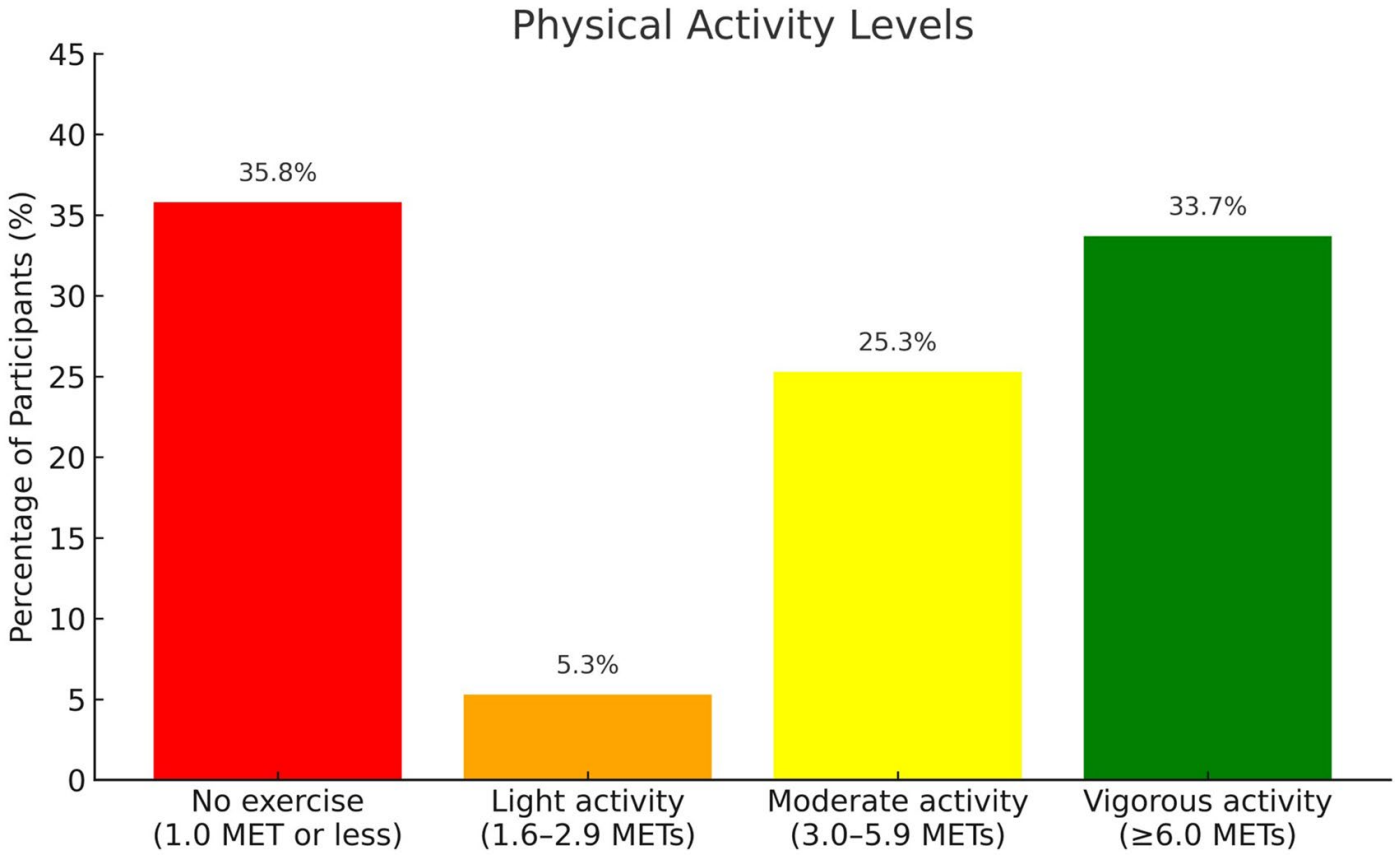
Distribution of self-reported physical activity levels. Bar chart showing the percentage of participants reporting each level of physical activity categorized by estimated metabolic equivalent tasks (METs): 35.8% reported no regular physical activity (≤1.0 MET), 5.3% reported light activity (1.6–2.9 METs), 25.3% reported moderate activity (3.0–5.9 METs), and 33.7% reported vigorous activity (≥6.0 METs).

The study cohort included 101 children and adolescents (36 boys) with multifactorial dyslipidemia. The mean age at body composition assessment was 13.8 ± 3.2 years. The cohort was characterized by a high socioeconomic position, with a median SEP cluster score of 8 [7, 9] (scores of 8–10 are considered high). Most were born at term (87.2%), and the median birth weight z-score was 0.16 [IQR 0.39, 0.91]. Physical activity data were self-reported. Overall, 64.2% of the cohort engaged in regular physical activity, with a median duration of 2 h per week [IQR 0, 3.8]. The distribution of activity intensity levels of none, light, moderate, and vigorous exercise is illustrated in Figure 1.

Anthropometric characteristics showed a mean height z-score of 0.28 ± 1.20, a median weight z-score of 2.09 [IQR 1.07, 2.54], and a median BMI z-score of 2.04 [IQR 1.47, 2.36]. At the time of referral, 17% of the cohort had a healthy weight, 16% were overweight, and 67% were obese. In terms of pubertal status, 17% were prepubertal, 32% were in puberty, and 51% were fully pubertal. The median systolic BP percentile was 90 [IQR 72, 96], and the median diastolic BP percentile was 76 [IQR 57, 87]. Body composition analysis revealed a median fat mass of 24.8 kg [IQR 17.1, 41.1], with a median fat mass z-score of 3.51 [IQR 1.45, 6.18]. The median trunk-to-appendicular fat ratio was 0.77 [IQR 0.69, 0.85]. The MFR ratio z-score was −1.62 [IQR –1.92, −1.24].

**Figure 2:**
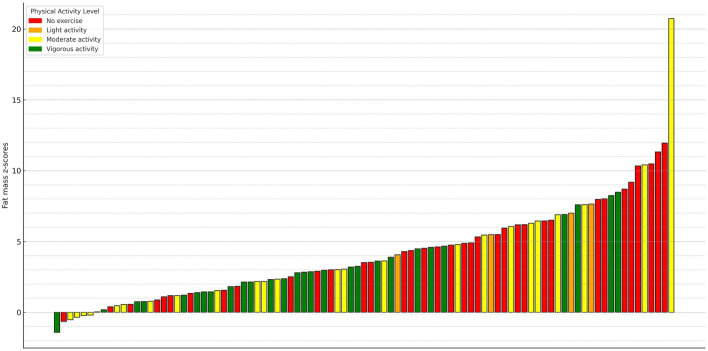
Waterfall plot of fat mass z-scores by physical activity level. Depicted are individual fat mass z-scores from lowest to highest. Each bar represents a single individual and is color-coded by physical activity level based upon standardized MET-based groupings: no exercise, light, moderate, or vigorous physical activity. This visualization highlights the distribution of adiposity across the physical activity spectrum.

Comparisons of anthropometric characteristics and body composition parameters stratified by fat mass z-score are presented in Table 1. There were no significant differences in sex distribution, age, or trunk-to-appendicular fat ratio between the groups. Individuals with a fat mass z-score ≥2 had significantly higher height, weight, and BMI z-scores, as well as elevated systolic and diastolic BPs (both absolute values and percentiles), compared to those with a fat mass z-score <2. The MFR ratio z-score was significantly lower in this group (all *p* < 0.001). A two-dimensional visualization of each individual’s physical activity level (METs) and corresponding fat mass z-score is shown in Figure 2. Physical activity levels were distributed across a wide range of fat mass z-scores, with no significant differences observed between groups (*p* = 0.169).

**Table 1:** Anthropometric and body composition characteristics by fat mass z-score (<2 vs. ≥2) in children with multifactorial dyslipidemia.

Variable	Fat mass z-score <2 *N* = 31	Fat mass z-score ≥2 *N* = 70	*p*-value
Sex, male	10 (32.3)	26 (37.1)	0.473
Age, years	13.1 ± 3.5	14.2 ± 3.2	0.112
Anthropometric parameters			
Height, z-score	−0.37 ± 1.12	0.66 ± 1.07	**<0.001**
Weight, z-score	0.63 [−0.14, 0.98]	2.35 [2.04, 2.67]	**<0.001**
Body mass index, z-score	0.99 [0.45, 1.28]	2.21 [2.02, 2.48]	**<0.001**
Blood pressure			
Systolic BP, percentiles	76 [58.3, 91.3]	94 [80, 97]	**<0.001**
Diastolic BP, percentiles	61 [43, 75]	80 [63, 89]	**<0.001**
Body composition parameters			
Fat mass, kg	14.8 [12.5, 17]	34.6 [24.5, 45.1]	**<0.001**
Trunk-to-appendicular fat ratio	0.79 [0.70, 0.86]	0.77 [0.68, 0.84]	0.770
Muscle-to-fat ratio, z-score	−1.05 [−1.36, −0.58]	−1.79 [−2.11, −1.51]	**<0.001**

Data are presented as *n* (%), mean ± standard deviation, or median [interquartile range]. Pearson’s chi-square test was used for categorical variables; the independent samples t-test or the Mann–Whitney U test was applied for continuous variables with normal or skewed distributions, respectively. Bold values indicate statistical significance at *p* ≤ 0.05. BP, blood pressure.

The median serum chemistry values were: glucose 88 mg/dl [IQR 83, 94], insulin 22.4 μU/ml [IQR 13.4, 30.8], glycated hemoglobin 5.4% [IQR 5.1, 5.6], CRP 0.25 mg/dl [IQR 0.22, 0.60], ALT 23 U/L [IQR 17, 37], and TSH 2.60 mIU/L [IQR 2.07, 3.85]. Acanthosis nigricans was observed in 46% of the individuals, and the median HOMA-IR was 5 [IQR 2.8, 6.8]. According to the pre-defined thresholds described in the Methods section, 100% of prepubertal children were classified as insulin resistant based on HOMA-IR, compared to 55% of pubertal and 52.9% of post-pubertal adolescents. The lipid profiles revealed a mean total cholesterol of 191.1 ± 33.1 mg/dl, a median triglyceride level of 195 mg/dl [IQR 145, 280], and a median HDL-C level of 40 mg/dl [IQR 37, 47]. The median non-HDL-C was 146 mg/dl [IQR 127.5, 162.5], and the mean LDL-C was 109.4 ± 30.1 mg/dl. The median TG:HDL-C ratio was 5.03 [IQR 3.31, 7.56].

**Figure 3:**
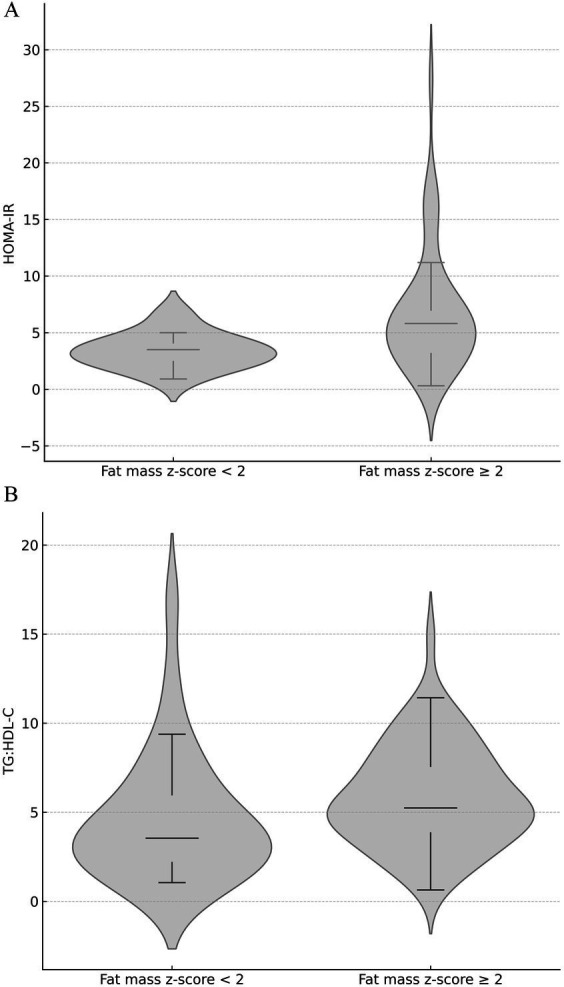
Hybrid violin and box plots illustrating the distribution and variability of cardiometabolic markers stratified by fat mass z-score category (<2 vs. ≥2) in children and adolescents. Panel **A** shows the distribution of HOMA-IR, with higher and more variable values observed in participants with fat mass z-score ≥2. Panel **B** shows the distribution of the TG:HDL-C ratio across the same fat mass z-score categories, demonstrating higher values in the elevated fat mass group.

Children and adolescents with a fat mass z-score ≥2 exhibited significantly higher levels of insulin, HOMA-IR, glycated hemoglobin, CRP, ALT, TSH, triglycerides, and the TG:HDL-C ratio compared to those with lower fat mass z-scores (Table 2). Figures 3a,b present the distributions of HOMA-IR and TG:HDL-C, respectively, stratified by fat mass z-score category. Both values were higher among individuals with a fat mass z-score ≥2, with a broader distribution and elevated medians compared to those with a fat mass z-score <2.

**Table 2:** Comparison of laboratory parameters between individuals with fat mass z-score (<2 and ≥2) in multifactorial dyslipidemia.

Laboratory parameters	Fat mass z-score <2 *N* = 31	Fat mass z-score ≥2 *N* = 70	*p*-value
Glucose, mg/dl	85 [83, 95]	88 [84, 94]	0.385
Insulin, mcU/ml	14.1 [12.5, 18.3]	27 [14.3, 33.3]	**0.008**
HOMA-IR	3.5 [2.5, 4.1]	5.8 [3.2, 7.0]	**0.015**
Glycated hemoglobin, %	5.3 [5.0, 5.4]	5.5 [5.2, 5.7]	**0.007**
C-reactive protein, mg/dl	0.25 [0.07, 0.25]	0.25 [0.25, 0.80]	**0.007**
ALT, U/L	19 [15, 23]	28 [21, 43]	**0.001**
TSH, mIU/L	2.43 [1.83, 3.29]	2.89 [2.12, 3.88]	**0.050**
TC, z-score	1.48 ± 1.24	1.39 ± 1.04	0.724
TG, z-score	1.79 [1.16, 2.92]	2.54 [1.96, 2.95]	**0.043**
HDL-C, z-score	−0.43 ± 1.08	−0.87 ± 0.95	0.055
Non HDL-C, z-score	1.79 [1.43, 2.29]	1.76 [1.12, 2.33]	0.668
TG:HDL-C ratio	3.55 [2.18, 5.98]	5.24 [3.84, 7.58]	**0.010**

Data are presented as *n* (%), mean ± standard deviation, or median [interquartile range]. Pearson’s chi-square test was used for categorical variables; the independent samples t-test or the Mann–Whitney U test was applied for continuous variables with normal or skewed distributions, respectively. Bold values indicate statistical significance at *p* ≤ 0.05. HOMA-IR, homeostatic model assessment for insulin resistance; ALT, alanine aminotransferase; TSH, thyroid-stimulating hormone; TC, total cholesterol; TG, triglycerides; HDL-C, high-density lipoprotein cholesterol.

Table 3 presents the correlation coefficients between body composition parameters and cardiometabolic markers, revealing a consistent pattern. Higher adiposity, as reflected by the BMI z-score, fat mass, and fat-mass z-score, showed moderate positive correlations with HOMA-IR (r ≈ 0.40–0.69), and weak positive correlations with both systolic and diastolic BP percentiles, TG z-scores, and the TG:HDL-C ratios (r ≈ 0.10–0.39), while only the fat-mass z-score reached a moderate positive correlation with diastolic BP percentiles. The BMI z-score alone displayed a weak negative correlation with the HDL-C z-score. The ratio between trunk and appendicular fat was not significantly correlated with any of the cardiometabolic markers. The MFR z-score showed weak negative correlations with both systolic and diastolic BP percentiles, HOMA-IR, and the TG:HDL-C ratio.

**Table 3:** Correlation between body composition parameters and cardiometabolic risk factors in multifactorial dyslipidemia.

	r	*p*-value
BMI, z-score		
Systolic BP, percentiles	0.362	**<0.001**
Diastolic BP, percentiles	0.328	**<0.001**
HOMA-IR	0.445	**<0.001**
TG, z-score	0.230	**0.022**
HDL-C, z-score	−0.252	**0.012**
TG:HDL-C	0.303	**0.002**
Fat mass		
Systolic BP, percentiles	0.337	**<0.001**
Diastolic BP, percentiles	0.386	**<0.001**
HOMA-IR	0.402	**0.002**
TG, z-score	0.223	**0.032**
HDL-C, z-score	−0.175	0.093
TG:HDL-C	0.267	**0.010**
Fat mass, z-score		
Systolic BP, percentiles	0.358	**<0.001**
Diastolic BP, percentiles	0.413	**<0.001**
HOMA-IR	0.425	**<0.001**
TG, z-score	0.209	**0.045**
HDL-C, z-score	−0.172	0.100
TG:HDL-C	0.267	**0.010**
Muscle-to-fat ratio, z-score		
Systolic BP, percentiles	−0.209	**0.046**
Diastolic BP, percentiles	−0.358	**<0.001**
HOMA-IR	−0.326	**0.013**
TG, z-score	−0.166	0.113
HDL-C, z-score	0.085	0.420
TG:HDL-C	−0.237	**0.023**

Spearman’s Rho correlation analysis was applied to determine the association between body composition parameters and lipid profile. All parameters were analyzed as continuous variables. BP, blood pressure; HOMA-IR, homeostatic model assessment for insulin resistance; TC, total cholesterol; TG, triglycerides; HDL-C, high-density lipoprotein cholesterol. Bold values denote statistical significance at p ≤ 0.05.

Self-reported physical-activity levels, expressed in METs, showed weak negative correlations with BMI z-scores (r = −0.231, *p* = 0.025), absolute fat mass results (r = −0.367, *p* < 0.001), fat-mass z-scores (r = −0.321, *p* = 0.002), and diastolic BP percentiles (r = −0.341, *p* < 0.001), and a weak positive correlation with the MFR scores (r = 0.223, *p* = 0.038). No significant correlations were observed between physical activity levels and HOMA-IR, TG z-score, HDL-C z-score, or the TG:HDL-C ratio.

Table 4 summarizes multivariable regression models for systolic and diastolic BP percentiles in children with multifactorial dyslipidemia. Models incorporating the BMI z-score demonstrated significant associations with both systolic and diastolic BP percentiles. After adjusting for sex, age, and physical activity levels, each one-unit increase in BMI z-score was associated with an 8.9 percentile increase in systolic BP (β = 8.910, *p* < 0.001) and a 9.0 percentile increase in diastolic BP (β = 8.961, *p* < 0.001). These models explained 14.6 and 17.3% of the variance (R^2^) for systolic and diastolic BP percentiles, respectively.

**Table 4:** Multivariable linear regression models evaluating explanatory factors for systolic and diastolic blood pressure percentiles in children and adolescents with multifactorial dyslipidemia.

Variable	β	SE	*p*-value	Lower bound for 95% CI	Upper bound for 95% CI	Adjusted R^2^
Systolic BP, percentiles						
Male	−7.661	4.328	0.080	−16.260	−0.938	0.108
Age	−0.098	0.634	0.877	−1.358	1.161	
Physical activity, hours	0.001	0.710	0.999	−1.410	1.412	
BMI, z-score	8.910	2.462	**<0.001**	4.019	13.801	
Diastolic BP, percentiles						
Male	−1.182	4.286	0.783	−9.697	7.334	0.136
Age	0.178	0.628	0.777	−1.069	1.425	
Physical activity, hours	−1.411	0.703	**0.048**	−2.808	−0.013	
BMI, z-score	8.961	2.438	**<0.001**	4.118	13.805	
Systolic BP, percentiles						
Male	−6.061	4.825	0.213	−15.659	3.536	−0.010
Age	−0.484	0.719	0.503	−1.915	0.947	
Physical activity, hours	−0.705	1.035	0.497	−2.763	1.352	
Fat mass, z-score	0.706	0.707	0.321	−0.701	2.113	
Diastolic BP, percentiles						
Male	−3.621	3.840	0.348	−11.258	4.017	0.270
Age	−0.782	0.572	0.176	−1.921	−0.356	
Physical activity, hours	−3.648	0.823	**<0.001**	−5.285	−2.010	
Fat mass, z-score	2.019	0.563	**<0.001**	0.900	3.138	
Systolic BP, percentiles						
Male	−12.414	5.248	**0.020**	−22.853	−1.975	0.067
Age	−0.382	0.673	0.572	−1.722	0.957	
Physical activity, hours	−0.125	1.025	0.903	−2.164	1.914	
MFR, z-score	−11.811	4.239	**0.007**	−20.244	−3.378	
Diastolic BP, percentiles						
Male	−9.871	4.189	**0.021**	−18.205	−1.538	0.319
Age	−0.324	0.537	0.548	−1.393	0.745	
Physical activity, hours	−3.054	0.818	**<0.001**	−4.682	−1.426	
MFR, z-score	−15.004	3.384	**<0.001**	−21.736	−8.273	

Independent variables included in each model: sex, age, physical activity levels (hours per week), and one explanatory variable (either the BMI z-score, fat mass z-score, or MFR z-score). Separate models were constructed for each explanatory variable. BP, blood pressure; MFR, muscle-to-fat ratio. Bold values denote statistical significance at *p* ≤ 0.05.

The fat mass z-score was significantly associated with diastolic, but not systolic, BP. Each one-unit increase corresponded to a 2.0 percentile increase in diastolic BP (β = 2.019, *p* < 0.001), with the model explaining 30.4% of the variance. Higher MFR z-scores were inversely associated with both systolic and diastolic BP percentiles. A one-unit increase in MFR z-score was associated with an 11.8 percentile decrease in systolic BP (β = −11.811, *p* = 0.007) and a 15.0 percentile decrease in diastolic BP (β = −15.004, *p* < 0.001). These models explained 11.0 and 35.1% of the variance in systolic and diastolic BP, respectively, with the latter representing the highest R^2^ for diastolic BP among all models examined.

Male sex was associated with lower systolic BP percentiles (β = −12.414, *p* = 0.020) and diastolic BP percentiles (β = −9.871, *p* = 0.021) in both models that included the MFR-z-score. In all three diastolic BP models, higher weekly physical activity was associated with lower BP percentile values, with each additional hour of reported activity corresponding to a 1.4–3.7 percentile decrease. No significant associations were observed between any of the variables and HOMA-IR or the TG:HDL-C ratio.

Additional analyses without adjustment for physical activity, preserving the total effect of body composition on blood pressure, yielded effect estimates highly similar to the primary models that adjusted for activity, with minimal attenuation (Supplementary Table 1). In the nested models (Supplementary Table 2), adding fat mass z-score to sex and age (Model B) produced a small change in explained variance (ΔR^2^ = 0.001, *p* = 0.209). Adding MFR z-score (Model C) increased the explained variance by ΔR^2^ = 0.056 (*p* = 0.009). In the full model, MFR remained independently associated with blood pressure (standardized β = −0.425, *p* = 0.009), whereas fat mass was not significant (β = −0.148, *p* = 0.339).

## Discussion

Our findings underscore the close interrelationship between excess adiposity, unfavorable lipid profiles, and early markers of cardiovascular risk among children and adolescents, reinforcing the importance of detailed phenotyping in this high-risk pediatric population.

The role of BMI in pediatric risk assessment is a subject of increasing debate given its limitations in accurately reflecting body composition and metabolic health. Assessment of body composition was shown to provide valuable insights, particularly among children with similar BMI values but divergent metabolic profiles. This level of granularity is particularly relevant in youth with overweight or obesity, where comparable anthropometric measures may obscure substantial variability in cardiometabolic risk. Our findings align with prior evidence of the limitations of BMI as a standalone metric and underscore the added value of incorporating detailed body-composition parameters into pediatric evaluations (13–15).

Consistent with previous reports demonstrating that greater fat mass is linked to elevated triglycerides regardless of muscle mass (33), our findings reinforce the central role of adiposity in shaping the atherogenic lipid profile characteristic of multifactorial dyslipidemia. Our results showed that increased fat mass and higher fat-mass z-scores were associated with elevated triglyceride levels, reduced HDL-C levels, and an increased dyslipidemic atherogenic index. This supports the utility of the TG:HDL-C ratio as a robust marker of cardiometabolic risk, in agreement with accumulating evidence suggesting that TG:HDL-C may outperform traditional lipid measures in predicting insulin resistance, hypertension, and other components of metabolic syndrome in youth (34–36).

Growing evidence highlights the muscle-to-fat ratio as a meaningful indicator of pediatric cardiometabolic health. Consistent with evidence from several observational studies (13, 14, 37), MFR in our cohort functioned as a surrogate marker of metabolic resilience in the context of excess adiposity. Although adiposity is widely recognized as the primary driver of cardiometabolic risk, our findings suggest that an imbalance between muscle and fat mass may also contribute to this risk. While both the fat mass z-score and the MFR z-score showed similar associations with triglycerides, HDL-C, the dyslipidemic atherogenic index, and blood pressure percentiles, the MFR provided additional context by reflecting the protective influence of muscle mass relative to fat. This interpretation is supported by the nested model analyses, which indicated that MFR captured body composition information more relevant to cardiometabolic risk than fat mass alone. These findings underscore the need to consider not only the extent of fat accumulation but also the balance between muscle and fat when assessing cardiometabolic risk in children.

Sarcopenic obesity in children and adolescents—defined by the combination of excess adiposity and insufficient muscle mass—is increasingly recognized as a clinically relevant phenotype associated with impaired metabolic flexibility, reduced physical fitness, and elevated cardiometabolic risk (38–42). Evidence consistently shows that a lower muscle-to-fat ratio is linked to adverse metabolic profiles, including higher inflammatory markers, liver enzymes, and blood pressure, as well as diminished muscular and cardiorespiratory fitness (39–42). Although no universally accepted definition exists and threshold values vary across studies (38, 43), the clinical literature supports sarcopenic obesity as a modifiable target. Interventions that promote gains in muscle mass—through resistance training, structured physical activity, adequate dietary protein, and healthy sleep patterns—alongside strategies to reduce excess adiposity have demonstrated potential to improve metabolic health in affected youth (40, 41, 44).

An unexpected finding in our cohort was that male sex was associated with lower systolic and diastolic blood pressure percentiles in multivariable models, which contrasts with most pediatric studies reporting higher blood pressure in boys than girls, even after adjusting for adiposity and other risk factors (45–48). This inverse association may reflect cohort-specific characteristics, such as sex differences in pubertal stage distribution, body composition patterns beyond those captured by our measured variables, or unmeasured behavioral and environmental factors. The sex imbalance in our sample (36 boys vs. 65 girls) may have also contributed to instability in sex-specific estimates. Given these findings deviate from established patterns, they should be interpreted cautiously and warrant replication in larger, more balanced cohorts.

In contrast, the trunk-to-appendicular fat ratio was not associated with any cardiometabolic outcome in our cohort, implying that regional fat distribution may be less critical than overall adiposity. This is contrary to the findings of several earlier studies in which the ratio outperformed BMI and total fat mass as a predictor of risk (49–51). Possible explanations include relatively homogeneous fat distribution among our participants, and the fact that regional fat was not directly measured but rather estimated from segmental BIA outputs. Although advanced imaging techniques, such as magnetic resonance imaging, provide more accurate differentiation between visceral and subcutaneous fat compartments, and have demonstrated stronger associations with cardiometabolic outcomes (52, 53), they are not routinely available or practical for use in most clinical settings. Consequently, while indirect measures of regional fat may offer some utility, their limitations must be acknowledged when interpreting risk in pediatric populations.

The more active children in our cohort displayed lower fat mass, lower BMI z-scores, lower diastolic BP, and higher MFR z-scores, emphasizing the protective role of regular physical activity in pediatric cardiometabolic health (54, 55). However, only two-thirds of the study participants reported engaging in regular physical activity, and the median duration was just 2 h per week—well below the 60 min per day of moderate-to-vigorous activity recommended by international guidelines (56, 57). This mirrors global surveillance data showing that most children and adolescents fail to meet activity targets (58), highlighting a modifiable public-health gap and reinforcing the need for interventions that make physical activity both accessible and enjoyable.

The uniformly elevated HOMA-IR values observed in the prepubertal children in our cohort indicate that insulin resistance can emerge well before the hormonal changes of puberty. Large prospective and cross-sectional studies similarly showed a steady rise in HOMA-IR with increasing adiposity, with overweight prepubertal children already exceeding the values of their healthy-weight peers, representing an early indication of the metabolic disturbances that precede dyslipidemia, hypertension, and other cardiometabolic complications (59–61). While HOMA-IR levels typically rise further during puberty due to physiological changes, our findings suggest that excess adiposity independently is associated with early insulin resistance. Moreover, elevated HOMA-IR may, in turn, promote adipose tissue dysfunction, lipid abnormalities, and vascular alterations, thereby creating a bidirectional cycle of cardiometabolic risk. This interplay underscores the need for early intervention to mitigate the long-term health impact of pediatric adiposity.

### Limitations and strengths

Our study has several limitations. First, the observational design precludes causal inference and is subject to selection and information biases. Physical activity was quantified as MET-hours per week based on reported activities classified using established MET intensity categories; however, sedentary time was not documented in medical records and could not be incorporated into analyses. The reliance on self-reported activity also introduces potential misclassification, which tends to attenuate true associations and may partly account for the absence of significant associations between physical activity and some metabolic outcomes (HOMA-IR, TG:HDL-C) in adjusted models. Second, although bioelectrical impedance analysis is practical and has been validated for pediatric use, it is less precise than gold-standard imaging methods such as bone density or magnetic resonance imaging for assessing regional body composition. Additionally, neither waist-to-height ratio nor skinfold thickness were measured, limiting the ability to evaluate fat distribution beyond impedance-based estimates. Third, the cohort included a higher proportion of girls than boys. Given known sex-related differences in growth, adiposity, and cardiometabolic physiology, this imbalance may limit generalizability, although adjustment for sex in multivariable models helps mitigate potential confounding. Finally, our cohort was drawn from a single tertiary center, which may restrict the applicability of these findings to broader pediatric populations. Our primary strength is the uniform clinical protocol, which included a standardized medical evaluation and BIA measurement, all performed by a single team of trained personnel.

## Conclusion

In this cohort of children and adolescents with multifactorial dyslipidemia, greater adiposity and lower MFRs were associated with elevated BP percentiles, insulin resistance, and atherogenic lipid profiles even in the prepubertal period. Incorporating standardized, BIA-derived body composition metrics into routine assessment improved risk stratification and revealed modifiable targets that may be missed by traditional measures, especially among inactive youth. This approach complements lipid screening by detecting early body composition abnormalities that may influence long-term cardiometabolic outcomes. Our findings support the integration of fat mass and the MFR into routine evaluation to better identify high-risk individuals and guide targeted interventions.

## Data Availability

The datasets presented in this article are not readily available due to ethical restrictions and the need to protect participant confidentiality. Access to anonymized data may be considered upon reasonable request and subject to approval by the Institutional Review Board of Tel Aviv Sourasky Medical Center. Requests to access the datasets should be directed to yaelleb@tlvmc.gov.il.
