# Supplementary material for: Body composition shapes cardiometabolic risk in children with multifactorial dyslipidemia
Source: Front Nutr. 2026 Jan 15;12:1717055. doi: 10.3389/fnut.2025.1717055 (PMC12851955; doi:10.3389/fnut.2025.1717055)
Supplement: Supplementary file 1 [file Table_1.docx]

| **SUPPLEMENTARY TABLE 1:** Multivariable linear regression models evaluating explanatory factors for systolic and diastolic blood pressure percentiles in children and adolescents with multifactorial dyslipidemia | | | | |
| --- | --- | --- | --- | --- |
| **Variable** | **β** | **SE** | ***P* value** | **R^2^** |
| Systolic BP, *percentiles* | | | |  |
| Male | -6.541 | 4.062 | **<.001** | 0.118 |
| Age | -0.291 | 0.599 | 0.629 |  |
| BMI *z-score* | 6.893 | 2.106 | **.001** |  |
| Diastolic BP, *percentiles* | | | | |
| Male | -1.097 | 4.062 | .788 | 0.136 |
| Age | 0.240 | 0.599 | .690 |  |
| BMI *z-score* | 8.072 | 2.106 | **<.001** |  |
| Systolic BP, *percentiles* | | | | |
| Male | -6.051 | 4.498 | .182 | 0.034 |
| Age | -0.406 | 0.687 | .556 |  |
| Fat mass *z-score* | 0.835 | 0.657 | .207 |  |
| Diastolic BP, *percentiles* | | | | |
| Male | -4.786 | 3.980 | .232 | 0.149 |
| Age | -0.440 | 0.608 | .471 |  |
| Fat mass *z-score* | 2.255 | .581 | **<.001** |  |
| Systolic BP, *percentiles* | | | | |
| Male | -10.393 | 4.682 | **.029** | 0.099 |
| Age | -0.405 | 0.640 | .528 |  |
| MFR *z-score* | -10.349 | 3.661 | **.006** |  |
| Diastolic BP, *percentiles* | | | | |
| Male | -10.399 | 4.011 | **.011** | 0.253 |
| Age | -0.106 | 0.548 | .847 |  |
| MFR *z-score* | -16.999 | 3.137 | **<.001** |  |
| Independent variables included in each model: sex, age, and one explanatory variable (either the BMI z-score, fat mass z-score, or MFR z-score). Separate models were constructed for each explanatory variable. Abbreviations**:** BP, blood pressure; MFR, muscle-to-fat ratio. **Bold** values denote statistical significance at  *p*≤ 0.05. | | | | |

| **SUPPLEMENTARY TABLE 2:** Nested regression models assessing the incremental contribution of fat-mass z-score and MFR z-score in children and adolescents with multifactorial dyslipidemia | | | | | |
| --- | --- | --- | --- | --- | --- |
| **Variable** | **Standardized β** | | ***P* value** | **R^2^** | **VIF** |
| Model A | | | | | |
| Male | -0.124 | | .248 | 0.017 | 1.037 |
| Age | -0.024 | | .825 |  | 1.037 |
| Model B |  | |  | |  |
| Male | -0.149 | | .174 | 0.018 | 1.070 |
| Age | -0.068 | | .544 |  | 1.150 |
| Fat mass *z-score* | 0.143 | | .209 |  | 1.169 |
| Model C | |  |  | |  |
| Male | -0.266 | | **.021** | 0.074 | 1.256 |
| Age | -0.034 | | .757 |  | 1.166 |
| Fat mass *z-score* | -0.148 | | .339 |  | 2.325 |
| MFR *z-score* | -0.425 | | **.009** |  | 2.454 |
| Model A includes age and sex. Model B adds fat-mass z-score to Model A. Model C adds MFR z-score to Model B. Standardized β coefficients and p-values are presented. ΔR² values represent the change in explained variance compared with the previous model. Variance inflation factors (VIF) from the full model are provided to assess multicollinearity; all values were <4, indicating no concerning collinearity. **Bold** values denote statistical significance at  *p*≤ 0.05. | | | | | |
